# Mechanistic Insight into How PEGylation Reduces the Efficacy of pH-Sensitive Liposomes from Molecular Dynamics Simulations

**DOI:** 10.1021/acs.molpharmaceut.1c00122

**Published:** 2021-06-06

**Authors:** Mohammad Mahmoudzadeh, Aniket Magarkar, Artturi Koivuniemi, Tomasz Róg, Alex Bunker

**Affiliations:** † Drug Research Program, Division of Pharmaceutical Biosciences, Faculty of Pharmacy, University of Helsinki, 00100 Helsinki, Finland; ‡ Medicinal Chemistry, Boehringer Ingelheim Pharma GmbH & Co. KG, Birkendorfer Strasse 65, D-88397 Biberach a.d. Riss, Germany; § Faculty of Pharmacy, University of Helsinki, P.O. Box 56, Viikinkaarie 5 E, FI-00014 Helsinki, Finland

**Keywords:** molecular dynamics simulations, PEGylated pH-sensitive liposomes, cholesteryl hemisuccinate, phase transition, bilayer hydrophilicity

## Abstract

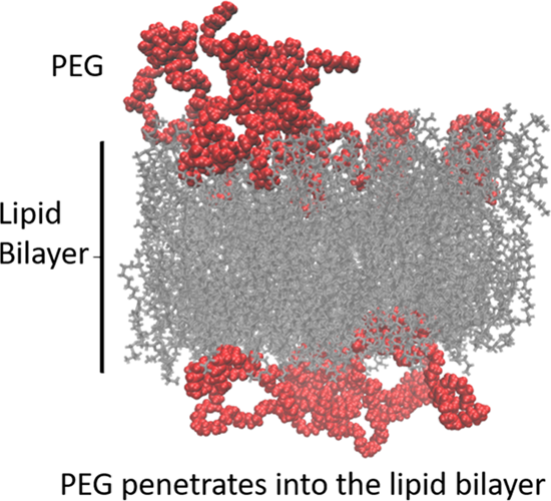

Liposome-based drug delivery systems composed of DOPE stabilized with cholesteryl hemisuccinate (CHMS) have been proposed as a drug delivery mechanism with pH-triggered release as the anionic form (CHSa) is protonated (CHS) at reduced pH; PEGylation is known to decrease this pH sensitivity. In this manuscript, we set out to use molecular dynamics (MD) simulations with a model with all-atom resolution to provide insight into why incorporation of poly(ethyleneglycol) (PEG) into DOPE–CHMS liposomes reduces their pH sensitivity; we also address two additional questions: (1) How CHSa stabilizes DOPE bilayers into a lamellar conformation at a physiological pH of 7.4? and (2) how the change from CHSa to CHS at acidic pH triggers the destabilization of DOPE bilayers? We found that (A) CHSa stabilizes the DOPE lipid membrane by increasing the hydrophilicity of the bilayer surface, (B) when CHSa changes to CHS by pH reduction, DOPE bilayers are destabilized due to a reduction in bilayer hydrophilicity and a reduction in the area per lipid, and (C) PEG stabilizes DOPE bilayers into the lamellar phase, thus reducing the pH sensitivity of the liposomes by increasing the area per lipid through penetration into the bilayer, which is our main focus.

## 1 Introduction

Lipid bilayers are of great importance in pharmaceutical nanotechnology. They can be formed into liposomes that can, in turn, be used as a nanoscale delivery system for small drug molecules,^1−4^ nucleic acids,^4−7^ and proteins.^8^ The dominant mechanism through which liposomes enter the cell is endocytosis, where they undergo enzymatic degradation by lysosomes.^9^ To disrupt this process, pH-sensitive liposomes have been proposed; in an acidic environment found within endosomes, they can destabilize the endosomal membrane, disrupting the formation of the lysosomal environment.^9^ pH-sensitive liposomes are formulated from a variety of lipid molecule types with the pH sensitivity achieved through several different possible mechanisms. For example, liposomes that have been designed to undergo a change in conformation in response to protonation/deprotonation^10−13^ become unstable through cleavage of the protective poly(ethyleneglycol) (PEG) corona due to chemical reactions induced by pH change,^14,15^ or, as proposed by Nahire et al., generate gas bubbles in response to low pH as a result of encapsulation by a precursor; the escaping gas bubbles disturb membranes and facilitate liposome content release.^16^ Change in the protonation state can increase drug solubility, thus triggering either release^17^ or transformation into a surfactant destabilizing liposome.^18^ Also, Mamasheva et al. constructed liposomes in which pH change triggers phase separation. Liposomes that have undergone phase separation remain stable but have increased permeability, and thus they slowly release their drug payload; the achieved controlled release can be advantageous in cancer therapy.^19^ Finally, Phoeung et al. demonstrated that large unilamellar vesicles composed of palmitic acid and cholesterol or cholesteryl sulfate released their content in response to pH changes.^20^ This case is particularly interesting as, in liposomes containing cholesterol, the protonation of palmitic acid occurs with a decrease in pH, triggering the release at low pH, whereas in the case of liposomes containing cholesteryl sulfate, the deprotonation occurs with increasing pH, triggering the release to occur at high pH.

Among all classes of pH-sensitive liposomes, the leading choice for the main phospholipid is phosphatidylethanolamine (PE).^21,22^ Preparation of stable bilayer vesicles from di-oleoyl PE (DOPE), at a physiological pH of above 10 °C, is however not possible^23^ unless either another lipid, e.g., phosphatidylcholine (PC), at greater than 30 mol % or an amphiphilic stabilizer with a carboxyl group is added.^24^ Cholesteryl hemisuccinate is the most popular choice in this regard,^25,26^ used extensively in the preparation of pH-sensitive liposomes in combination with DOPE. Although stable DOPE liposomes can only be made at high pH (>9.0) where PE is negatively charged,^27^ incorporation of cholesteryl hemisuccinate (CHMS) into DOPE liposomes stabilizes them into the lamellar phase at a physiological pH of 7.4;^28^ at 25 °C, the addition of CHMS in excess of 20 mol % stabilizes DOPE vesicles.^29^ The structure of the CHMS molecule includes a carboxylic acid group with a p*K*_a_ of 5.8; its ionic state determines the phase behavior of the DOPE ensemble.^28^ At physiological pH, the vast majority (98%) of CHMS is in the deprotonated (anionic) form^30^ and thus is negatively charged (in this article, we refer to the anionic form of CHMS as CHSa); in an acidic environment, CHMS is protonated and thus becomes neutral (in this article, we refer to the neutral form of CHMS as CHS).

Although there is general agreement that CHSa stabilizes DOPE bilayers through a change in phase behavior of the lipid ensemble, several different mechanisms have been proposed for how the bilayer is stabilized. Lai et al. proposed that the membrane-stabilizing effect of CHSa is probably due to the disruption of the intermolecular interaction between adjacent PEs,^29^ whereas Torchilin et al. attributed the CHSa stabilizing effect to its ability to increase liposomal interfacial hydration.^31^ Yet other studies consider the electrostatic repulsion provided by CHSa as the stabilizing mechanism.^28^

The protonation of CHMS (transformation from CHSa to CHS) leads to the rapid destabilization of liposomes; CHS lacks the membrane-stabilizing properties of CHSa. It is noteworthy that cholesterol (CHOL) also decreases the stability of the 1-palmitoyl-2-oleoyl-phosphatidylethanolamine (POPE)/CHOL lamellar phase when its concentration in the lipid mixture is increased from 0 to 30%. Interestingly, a further increase of the CHOL concentration reversed this effect.^32^ However, the atomistic details leading to the destabilization of CHMS/DOPE lamellar phases are not clearly understood. This is due to the inherent complexity of the process, which involves the formation of intermediate structures; it is known that with a reduction in pH, DOPE–CHSa transforms into DOPE–CHS, leading to liposomal aggregation and inter bilayer contact and ultimately disruption of the liposomes.^27^ The contact-induced destabilization does not, however, lead to any mixing of aqueous contents of liposomes;^33^ it only causes lipid mixing at pH of 5 or below.^34^

Liposomes are successful drug delivery systems in vitro; however, their application is hindered by rapid clearance from the blood stream following injection. To increase the circulation half-life of liposomes, they are sterically stabilized by PEGylation through incorporating PEGylated lipids into the liposome formulation.^1,2,21^ Although PEGylation significantly increases the circulation half-life of liposomes, it also unfortunately causes a dramatic reduction in pH sensitivity. DOPE–CHMS liposomes release 55% of their loaded calcein at a pH of 5.5, whereas PEGylated DOPE–CHMS liposomes with 0.6 mol % DSPE-PEG2000 release only 24% and only 10% in the case of 3 mol % DSPE-PEG2000.^35^ Slepushkin et al. also reported that when 5 mol % DSPE-PEG is incorporated into DOPE–CHMS liposomes, the release of calcein in buffer, with pH 5.5, decreases from 84 to 6.8%.^36^

It has been experimentally proven that a lamellar-to-hexagonal phase transition occurs following the change of CHSa into CHS.^27,33,34^ Although it is not practically feasible to investigate the whole process of this phase transition using all-atom MD simulations, large-scale simulations of lipid systems already in the hexagonal phase have been carried out for DOPE, POPE,^37^ and galactolipids,^38^ and using coarse-grained simulations, the phase transition of DOPE lipids from lamellar to hexagonal has been modeled;^39^ it is however not possible to investigate the effect of PEGylation on this transition as current coarse-grained models of PEG fail to reproduce the relevant properties of PEG. In this study, using all-atom MD simulations, our focus is to elucidate the effect of charged and neutral forms of CHMS on the properties of DOPE lipid bilayers with and without PEGylation. This information helps to understand the atomistic details governing the change of stability and consequently the pH sensitivity of DOPE/CHMS liposomes upon CHMS protonation and the effect of PEGylation on this, which is the primary focus of this work. Despite the crucial role of CHMS in the formation and function of pH-sensitive liposomes, as of yet, no computational simulations have been conducted on DOPE bilayer containing CHMS. The only published MD simulations of CHMS in lipid membranes are the two works performed by Kulig et al. concerning the similarity of the behavior of CHMS and cholesterol in saturated and unsaturated PC bilayers.^30,40^

During the last 10 years, our group has conducted MD simulations on lipid bilayers and PEGylated liposomes,^41−47^ where the effect of the presence of PEG, its grafting density, molecular weight, and conformation on structural characteristics and behavior of the membrane have been evaluated in detail. We now continue this line of research; in this publication, we shed light on the reason why PEGylation reduces the pH sensitivity of DOPE–CHMS liposomes. We have additionally addressed two other questions: (1) how CHSa stabilizes DOPE bilayers in lamellar conformation at a physiological pH of 7.4 and (2) how the change from CHSa to CHS at acidic pH triggers the destabilization of DOPE bilayers; to this end, we have conducted MD simulations using six model systems as summarized in Table 1.

**Table 1:** Detailed Description of the Composition of All of the Six Simulated Systems

system abbreviation	DOPE	CHOL	CHS	CHSa	PEG	Na^+^	Cl^–^	K^+^	water	MD length (ns)
DOPE–CHOL	192	96	0	0	0	30	30	0	12 140	300
DOPE–CHS	192	0	96	0	0	30	30	0	12 140	300
DOPE–CHSa	192	0	0	96	0	30	30	96	12 044	300
PEG–DOPE–CHOL	192	96	0	0	14	70	70	14	30 144	400
PEG–DOPE–CHS	192	0	96	0	14	70	70	14	30 144	400
PEG–DOPE–CHSa	192	0	0	96	14	70	70	110	30 048	400

## 2 Methods

### 2.1 System Setup

**Figure 1:**
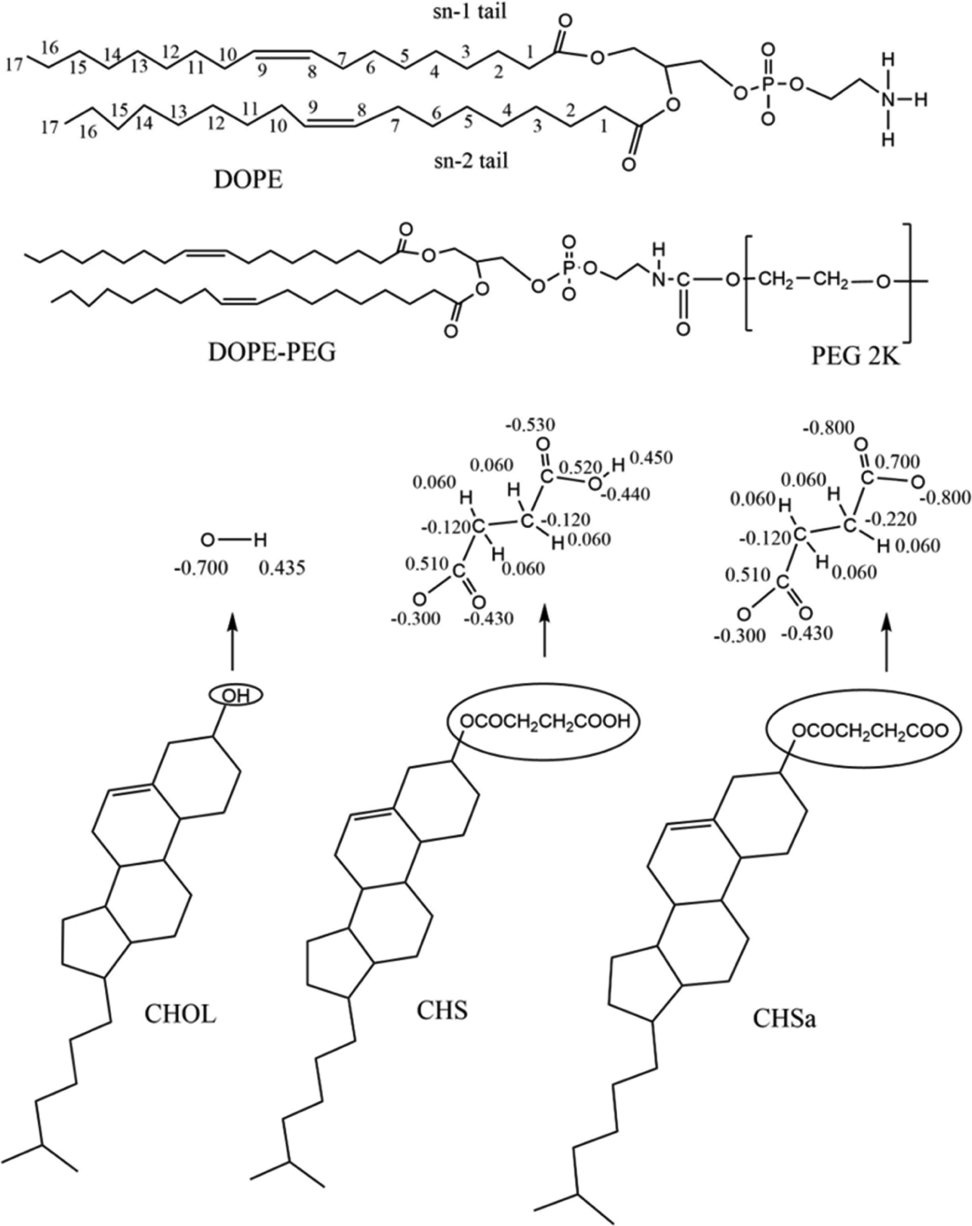
Chemical structure of the components of the bilayer showing atom numbering for the acyl chains of DOPE and the partial charges derived for CHOL, CHS, and CHSa.

We have conducted MD simulations on six systems. Three of them are non-PEGylated, where the bilayer is composed of 192 DOPE phospholipids (we refer to phospholipids as lipids in this article) and 96 steroid molecules: cholesterol (CHOL), CHS, or CHSa. In the other three simulated systems, the bilayer is composed of 192 lipids, 14 of which are PEGylated (PEGylated DOPE lipids) and 96 of which are steroid molecules. The detailed description of the components for all six systems is shown in Table 1, and their chemical structures are displayed in Figure 1. The membranes were constructed using an in-house python script. This script creates a randomized organization of provided lipids and steroid molecules based on their proportions in a bilayer format. Following solvation, in all systems, 125 mM NaCl was added and K^+^ counter ions were used where necessary to obtain charge neutrality for all systems. Before starting the MD simulations utilizing a Gromacs 4.6.7 software package,^48,49^ the starting configurations of the lipid bilayers were energy minimized using the steepest descent method to remove any possible bad contacts among the atoms and then simulated for 100 ns with position restraints on the lipid head group and tail C atoms with a force constant of 1000 kJ/(mol nm^2^) in the *Z* direction. This was followed by a 200 ns simulation without restraints to relax the membrane for obtaining the starting configuration of the membranes. Since the liposomes have a diameter of ∼100 nm and the box dimension of our simulated slab of the bilayer is ∼8 nm, curvature effects can be assumed to be negligible.

### 2.2 Molecular Model Parametrization

We used the all-atom optimized parameters for the liquid simulations (OPLS-AA) force field^50,51^ for the parametrization of the steroids, PEG, and ions. For the lipids, in addition to the OPLS-AA force field, we used additional parameters from the force field we recently developed, specially for lipids compatible with the OPLS-AA force field.^52−54^ For water, we employed the compatible TIP3P model.^55^

### 2.3 Molecular Dynamics Simulation Parameters

For all MD simulations, the pressure was controlled at a constant pressure of 1 bar using the Parrinello–Rahman barostat^56^ with a pressure coupling constant of 1 ps and a compressibility of 4.5 × 10^–5^ bar using a semi-isotropic pressure scheme. The temperatures of the solute and solvent were set to 310 K, controlled independently using the Nose–Hoover thermostat.^57,58^ Periodic boundary conditions, with the usual minimum image convention, were used in all three directions (*x*, *y*, and *z*). The Linear Constraint Solver (LINCS) algorithm^59^ was used to preserve covalent bond lengths, and the simulation time step was set to 2 fs. The Lennard–Jones interactions were calculated within a 1.0 nm cutoff; for the electrostatic interactions, we employed the particle mesh Ewald method.^60,61^

### 2.4 Analysis

We performed all of the analysis on the last 100 ns of the trajectory; monitoring of the area per molecule assured us that all systems had reached equilibrium. We calculated the averaged area per molecule by dividing the total area of the simulation box in the *x*–*y* plane by the number of all molecules (lipids and steroids) in a single leaflet and then averaging it over all of the frames of the last 100 ns of the trajectory.

Following the calculation of the area per molecule, we continued our analysis with visualizations of the systems using visual molecular dynamics (VMD)^62^ to obtain an intuitive picture of the behavior and interactions of the components of the simulated systems. The solvent-accessible surface area (SASA) value for the hemisuccinate moieties of CHS and CHSa was measured over the course of the simulation time with a solvent probe radius of 1.4 Å, using analysis tools found within the VMD software package. To investigate the interactions of the ions with both the lipid bilayers and PEG, the percentage of Na^+^ ions in contact was calculated; a contact was considered to exist when the distance between any pair of atoms from the respective groups was equal to, or less than, 0.325 nm.^63^

To estimate the extent of lipid chain ordering, we then measured the average values of the deuterium order parameters (|*S*_CD_|) for the lipid acyl chains given by
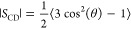
1where θ is the angle between the C–D bond and the bilayer normal, and ⟨...⟩ denotes averaging over time and all lipid molecules;^64^*S*_CD_ was calculated using the gmx_order tool included in the Gromacs simulation package.

Finally, the mass density profile perpendicular to the membrane normal for PEG oxygen atoms, lipids, and water molecules was calculated for each system utilizing the gmx_density tool of the Gromacs package. Mass density profiles of different systems were shifted such that the distributions of the phosphate groups overlap; this allows for a direct comparison of the depth of penetration of water and PEG into the bilayer.

## 3 Results

### 3.1 Area Per Molecule

**Figure 2:**
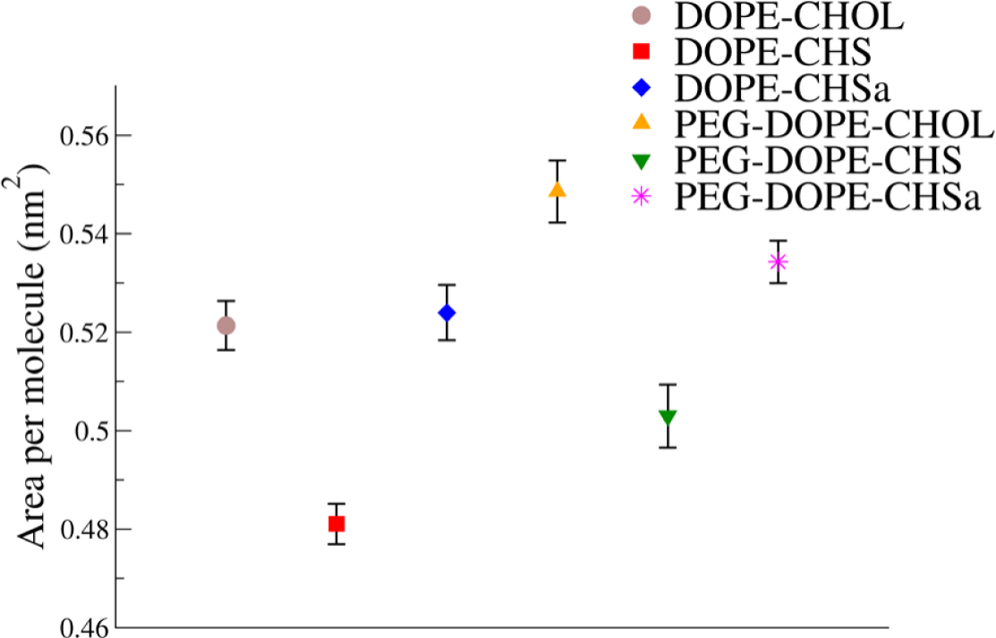
Average area per molecule.

The effect on the area per molecule resulting from alteration of the steroids in the formulation and the addition of PEG was determined. Averaged values of the area per molecule for all systems are shown in Figure 2. Replacement of CHOL with CHSa does not affect the area per molecule in the absence of PEG and slightly decreases the area in the presence of PEG. When CHSa changes to CHS, the area per molecule decreases.

### 3.2 Membrane Hydration

**Figure 3:**
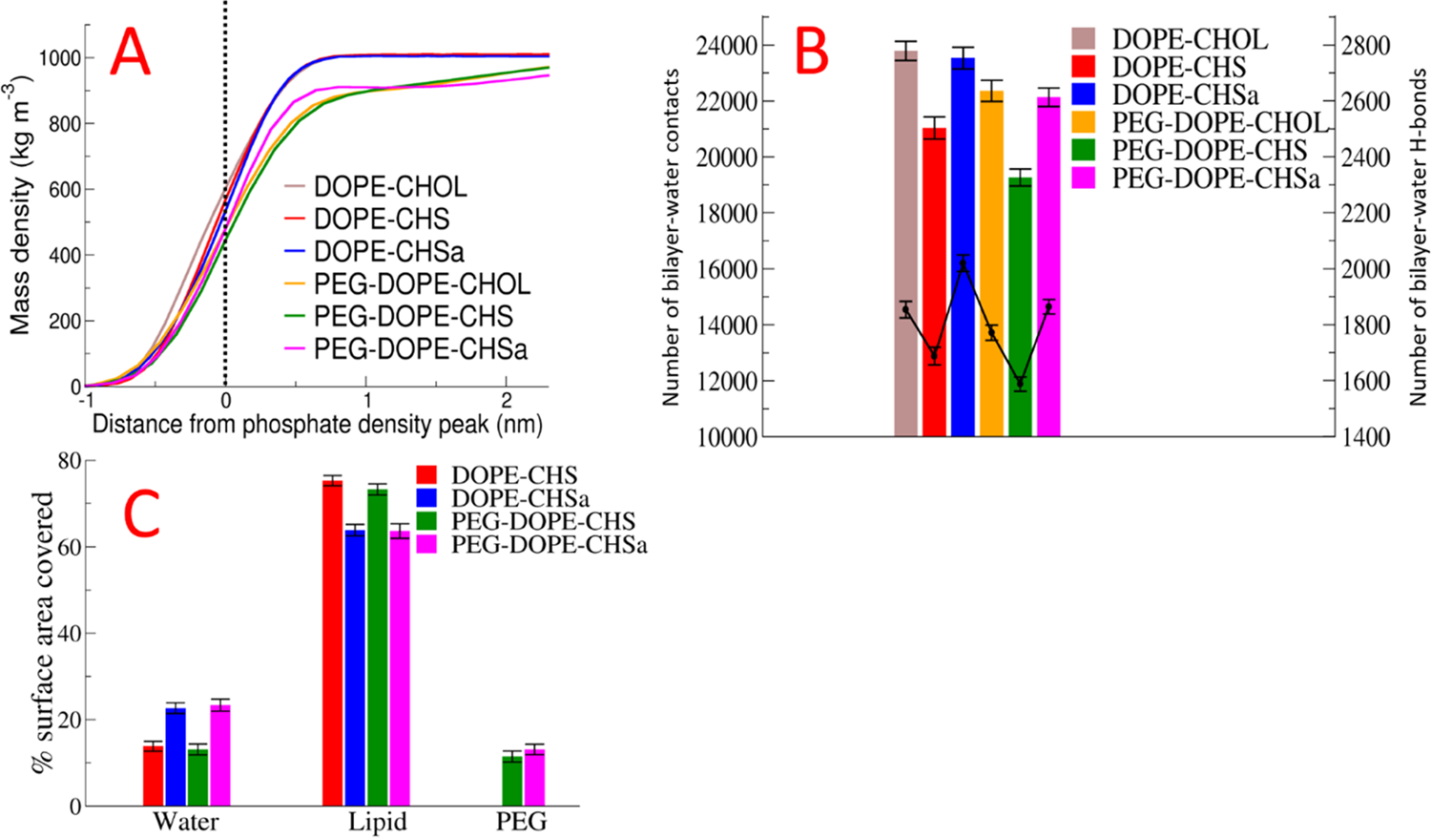
Partial mass density profiles of water relative to the position of the phosphate head group peak (A). Number of bilayer–water contacts (shown in bar graphs with values on the left-hand side *Y*-axis) and number of bilayer–water H-bonds (in line graphs with values on the right-hand side *Y*-axis) (B). Percentage of area of the steroid head groups covered by other components of the system (C).

In Figure 3A, we show the mass density profiles for water relative to the position of the phosphate head group. Two clear trends are observed: water penetration is the deepest for the bilayers with CHOL, and PEGylation is seen to reduce the degree of water penetration. In agreement with our observations regarding the depths of water penetration into the bilayer and area per molecule, the number of bilayer–water contacts is similar in the bilayers with CHOL and CHSa and reduced in the bilayers with CHS (Figure 3B). Next, PEGylation decreases the number of water–bilayer contacts and H-bonds in all studied models (Figure 3 B). In agreement with all of the above results, the SASA was found to be larger for CHSa than that for CHS (Figure 3C). The observed differences between bilayers containing CHSa and CHS result from the charged nature of CHSa, the more polar form of the molecule. The decreased hydration of the lipids in bilayers containing PEGylated lipids (Figure 3A,B) results simply from direct coverage of lipids by PEG (Figure 3C). This result is in agreement with prior studies by Tirosh et al., who reported that grafting PEG to liposomes at 5–7 mol %, will decrease hydration of the lipid head group.^65^

### 3.3 Number of Intra-Bilayer H-Bonds

Table 2 shows the numbers of lipid–lipid, lipid–steroids, and lipid–PEG H-bonds. The number of H-bonds for the DOPE–CHOL bilayer, both DOPE–DOPE and DOPE–CHOL, is lower than that of the DOPE–CHS/CHSa bilayers. The number of sterol–lipid H-bonds in DOPE–CHSa is more than doubled in comparison to that in DOPE–CHOL; this probably acts to compensate for the electrostatic repulsion force between the CHSa head groups, preventing an increase in area per molecule in DOPE–CHSa in comparison to DOPE–CHOL.

**Table 2:** Number of Bilayer–PEG, Interlipid, and Lipid–Steroid H-Bonds

system name	DOPE–DOPE	DOPE–steroid	bilayer–PEG
DOPE–CHOL	78.82 ± 8.91	32.95 ± 4.56	
DOPE–CHS	91.02 ± 7.92	57.52 ± 4.95	
DOPE–CHSa	93.08 ± 8.37	75.83 ± 5.43	
PEG–DOPE–CHOL	81.11 ± 7.28	33.74 ± 4.20	13.25 ± 6.08
PEG–DOPE–CHS	92.38 ± 8.17	56.82 ± 5.03	26.71 ± 4.10
PEG–DOPE–CHSa	88.86 ± 7.72	66.51 ± 5.66	14.71 ± 3.13

PEGylation does not affect the extent of H-bond formation between DOPE–DOPE, DOPE–CHOL, and DOPE–CHS; however, it reduces the number of H-bonds between DOPE and CHSa. Interestingly, the number of lipid–PEG H-bonds is two times greater in the PEG–DOPE–CHS bilayer in comparison to the two other bilayers.

In Section 3.1, we have already demonstrated that the area per molecule in DOPE–CHOL and DOPE–CHSa bilayers are similar, while in DOPE–CHS, it is smaller. This result seems to be in disagreement with prior studies on DOPC and DPPC, where the effects of CHOL on membrane properties were stronger than those of CHS and CHSa.^30,40^ However, for the case of DOPE, the number of steroid–DOPE H-bonds is doubled for the case of CHSa/CHS in comparison to that for CHOL. This probably explains the observed difference between PC- and PE-based bilayers.

### 3.4 Order Parameter and Lipid Mass Density Profiles

**Figure 4:**
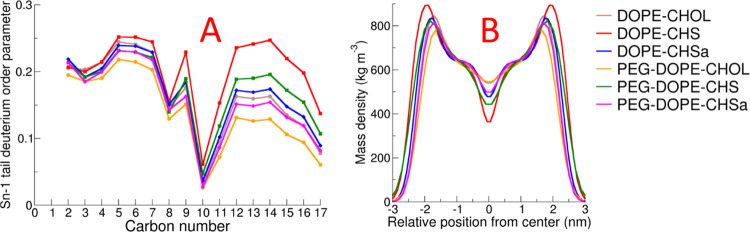
Deuterium order parameter along the Sn-1 tail (A) and mass density profile results for the lipid component (B).

Figure 4A shows the profile of the deuterium order parameter along the Sn-1 acyl chains of the lipids. The deuterium order parameter is related to the surface area of the lipids through a direct inverse relationship.^64^ A higher deuterium order parameter generally means a more compact bilayer. The most ordered lipid tails are observed in bilayers containing CHS, which are also characterized by the smaller surface area per lipid. In all cases, the lipid tail ordering was observed to be reduced by PEGylation.

Figure 4B shows the mass density profiles of the lipids in the simulated systems. The mass density profile shows the membrane thickness, also related to the order parameter; when the extent of lipid ordering is increased, the bilayer becomes thicker and density increases, and thus the value of the density in the profile will increase. This trend is present in the data shown in Figure 4B. In non-PEGylated bilayers, the density has the highest value and the bilayer is thickest for the DOPE–CHS membrane; in the DOPE–CHOL and DOPE–CHSa bilayers, the density is reduced and the bilayer is thinner. The presence of PEGylated lipids decreases the density and the bilayer thickness, though this effect is small for the case of the DOPE–CHSa bilayer.

### 3.5 Ion Contacts with Bilayer and PEG

**Figure 5:**
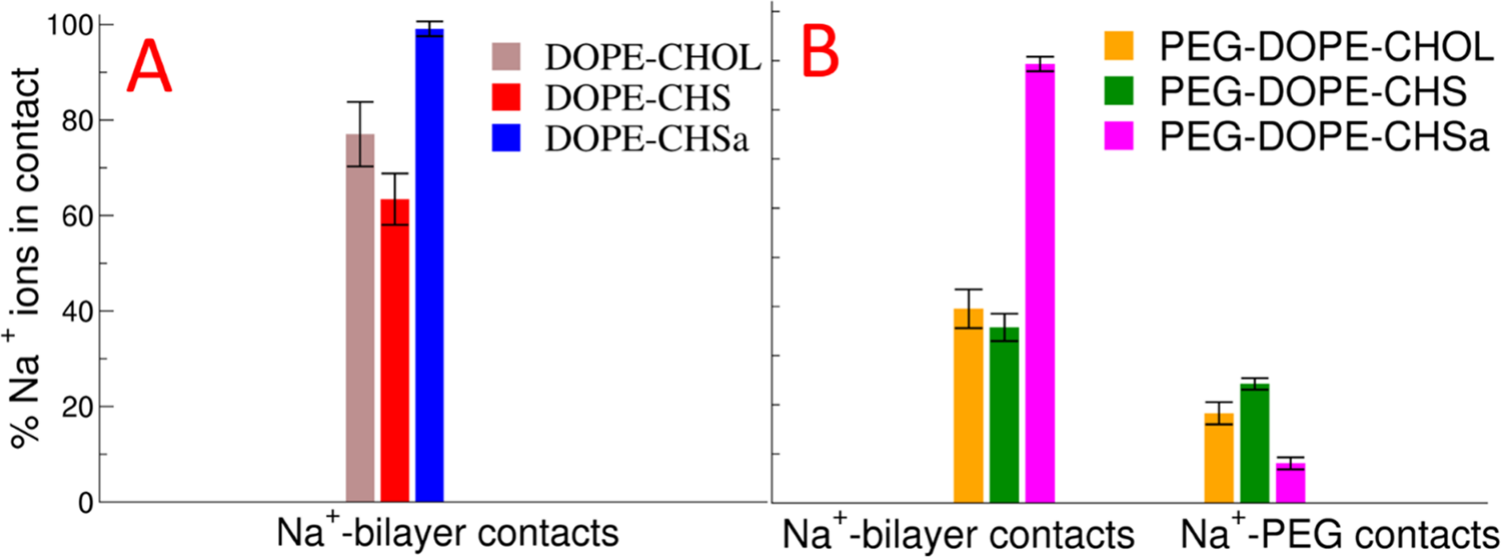
Percentage of Na^+^ ions in contact with the bilayers in non-PEGylated systems (A) and percentage of Na^+^ ions in contact with the bilayers and PEG chains of the PEGylated systems (B).

We next calculated the contacts of the Na^+^ ions with both the bilayers and PEG (Figure 5). Figure 5 shows two clear trends; first, in bilayers containing CHSa, the ion binding of Na^+^ is the strongest, and second, the presence of PEGylated lipids decreases the extent of Na^+^ binding. The effect of the presence of PEG on Na^+^ binding is strongest in the bilayers with neutral steroids and weakest in the bilayers containing CHSa. Adsorption of Na^+^, K^+^, or arginine due to deprotonation of CHMS was observed in a prior MD simulation study.^66^

The results of this section must be interpreted with caution as recent studies of PC bilayers have shown that the extent to which cations bind to a neutral lipid bilayer is severely overestimated.^67^ Thus, we can expect similar problems concerning PE lipids; however, in comparison to DOPC, we observed a reduced number of ions adsorbing to the water membrane interface of DOPE bilayers. Conversely, negatively charged lipids are expected to bind cations, including monovalent cations,^68,69^ although, as far as we are aware, no experimental studies on CHSa have so far been carried out.

### 3.6 PEG Penetration into Lipid Bilayers

**Figure 6:**
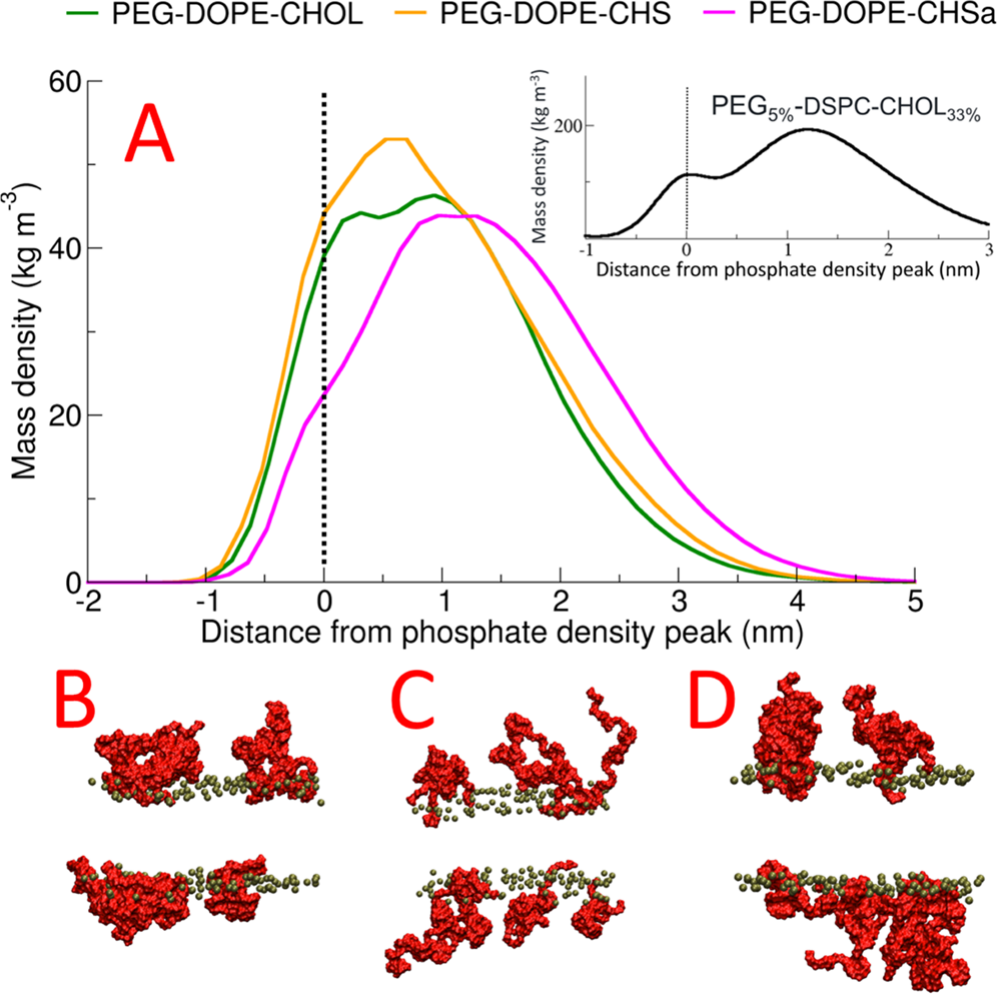
Mass density profile of PEG relative to the position of the phosphate head group peak (A) and visualization of the final configurations of the system in the last frame of the simulation of PEG–DOPE–CHOL (B), PEG–DOPE–CHS(C), and PEG–DOPE–CHSa (D). Phosphate atoms are colored as green and PEG chains as red. All other components of the systems were removed for clarity. The inset in Figure 6A represents the PEG mass density profile of PEG_5%_–DSPC–CHOL_33%_ studied in our previous publication (ref (44)).

We next calculated the mass density profiles for the PEG polymer in all systems, along the bilayer normal. We observed a striking difference between the behavior of PEG in DOPE bilayers (see Figure 6) and that of the DSPC with the cholesterol bilayer (Doxil formulation) studied in our previous publication;^44^ for the case of the PC system, PEG is predominantly located outside the bilayer with a clear, smaller, secondary peak completely inside the bilayer core and a reduced density of PEG between these two locations at the position of the lipid head groups (inset in Figure 6A). In the case of the DOPE bilayers, in contrast, we do not see the same two-peak distribution, but rather a single peak at roughly the position of the phosphate head group; PEG locates to the position of the head groups to a far greater extent (see Figure 6). The behaviors of the three DOPE systems are qualitatively similar; however, the extent of penetration into the membrane is decreased for the system with CHSa in comparison to the others. This observed penetration of PEG into the DOPE bilayer can be seen as the cause of the significantly lower lipid tail order parameter value for PEGylated DOPE systems in comparison to non-PEGylated DOPE systems that we observed (Figure 4A).

## 4 Discussion

To understand why PEGylation reduces the sensitivity of pH-sensitive liposomes and, additionally, how CHSa stabilizes DOPE bilayers and how the change from CHSa to CHS destabilizes them, we have conducted MD simulations of DOPE systems containing CHOL, CHS, and CHSa with and without PEG. Since the stabilization and destabilization of the DOPE ensemble result from the phase transition, using our results and those obtained by experimental evaluations, we attempt to justify this observed behavior in the context of lipid hydration and the dynamic molecular shape theory applied to lipids, introduced by Cullis et al.^70^

Replacing PC with PE in the lipid head groups of a membrane is known to increase the hydrophobicity of the membrane surface,^71^ thus resulting in an increased tendency for DOPE bilayers to undergo a lamellar-to-hexagonal phase transition.^31^ The low hydration extent of DOPE is related to the small size of the DOPE head group and formation of intermolecular H-bonds between amine and phosphoryl groups of adjacent DOPE molecules.^31^ The network of H-bonds plays an important role in the phase behavior of DOPE lipids;^72^ H-bonds between adjacent DOPE molecules replace some of the PE–water H-bonds,^73^ which diminishes water penetration in the polar head group region and reduces the polarity of the PE head groups.^71^ Based on our results, CHSa stabilizes the DOPE lipid membrane through increasing the hydrophilicity of the bilayer surface, and as we can see in Figure 3B, the number of bilayer–water H-bonds is significantly greater for the systems with CHSa than those with CHOL. When CHSa changes into CHS, the bilayer surface is dehydrated and a large reduction in the number of bilayer–water H-bonds is observed.

In addition to the head groups, as the polar components of the bilayer, it has been experimentally proven that the packing of the lipid chains would affect the extent of lipid hydration.^74^ In our simulations, we observed that DOPE–CHS, with the highest extent of lipid tail ordering among non-PEGylated systems, has the lowest extent of bilayer–water H-bonds in comparison to the other non-PEGylated systems.

The ability of lipids to exist in various phases can be explained by dynamic molecular shape theory; there are three varieties of shapes available for lipids based on the ratio of the cross-sectional area of the acyl chain to the area of the head group. Due to its small head group and bulky acyl chains, DOPE is considered as a cone-shaped lipid,^31^ which forms non-bilayer phases, for example, the hexagonal (HII) phase. Cylindrical-shaped lipids like PC have a similar cross-sectional area of the head group and acyl chains and form lamellar phases. Inverted cone-shaped lipids have a larger head group in comparison to the acyl chains and tend to form micelles in aqueous solutions.^23^

Regarding the concept of the dynamic molecular shape of lipids, stabilization of cone-shaped lipids in the bilayer phase is possible by either reducing the ratio of the cross-sectional area of the acyl chain to the area of the head group^24^ or mixing cylindrical lipids like PC or inverted cone-shaped lipids like PEGylated lipids with cone-shaped lipids.^23^ Reducing the ratio of the cross-sectional area of acyl chains to the area of the head group is possible through increasing the area of the head group either by (1) decreasing the intermolecular interaction of the head groups, which inhibits the lateral expansion of the bilayer,^24,73^ (2) binding cations with the head group,^66^ or (3) increasing the extent of the hydration of the head group.^75,76^ It is also possible to reduce the cross-sectional area of acyl chains by increasing their order by, e.g., adding cholesterol.^77,78^ On the other hand, an increase in temperature will increase the ratio of the cross-sectional area of acyl chains to the area of the head group, leading to a phase transition from the lamellar phase to the hexagonal phase.^79^ Also, low hydration is a factor promoting the formation of hexagonal phases.^80^ Finally, vitamin E (α-tocopherol) promotes formation of the hexagonal phase by an alternative, more complex mechanism.^81^

In our simulations, we attempt to determine how the change of CHSa to CHS triggers the destabilization of DOPE bilayers. We observed that when DOPE–CHSa changes to DOPE–CHS, a reduction in the head group area occurs, which, in turn, based on molecular dynamic shape theory, could possibly facilitate the phase transition of DOPE bilayers from the lamellar phase to the hexagonal phase. This reduction in the area per lipid occurs due to the reduction in the number of bound Na^+^ ions and water molecules hydrating the bilayer (Figures 3B and 5). Our results agree with the prior studies of Klasczyk et al., who conducted MD simulations on bilayers composed of only CHSa; they observed all cations in the simulation to be adsorbed by the CHSa. Following this observation, they emphasized the importance of dynamic molecular shape theory and attributed the stabilization of the CHSa bilayer to the increase in the effective head group volume of CHSa due to cation binding to its head group.^66^

In our simulations, we also observed the DOPE–CHSa bilayer to adsorb all of the Na^+^ ions in the solution, while DOPE–CHS adsorbs 60% of the Na^+^ ions. These numbers are likely overestimated due to the overbinding of Na^+^ ions to the lipid head groups; this results from the lack of explicit polarizability in the force field.^67^ Thus, this remains a qualitative observation that indicates a large difference in the behavior of the two systems; however, the binding of cations to bilayers containing negatively charged lipids is supported experimentally.^68,69,82^ Additionally, simulations performed by Klasczyk et al. indicated that CHSa attracts and binds counter ions while CHS did not.^66^ These simulations were performed with the GROMOS87 force field, which seems to be less affected by the lack of explicit polarizability.

Based on our results, PEG stabilizes DOPE–CHMS liposomes by increasing the area per lipid which, based on molecular dynamic shape theory, prevents the phase transition of the DOPE bilayers from lamellar to hexagonal phase. We observed significant penetration of PEG into the DOPE bilayer; this penetration was greater than the penetration of PEG into the bilayers composed of PC lipids that we studied in our previous works.^2,42−45^ This effect can be attributed to the high number of bilayer–PEG H-Bonds that cannot occur in the case of PC lipids as a result of the lack of H-bond donors in both PEG and the PC head groups. This observation is in agreement with the work of Holland et al., who reported that the addition of PE–PEG to a mixture of DOPE–CHOL that adopts a hexagonal phase when hydrated under physiological conditions stabilizes the mixture in a bilayer conformation.^23^ They explained their observation in terms of the dynamic molecular shape concept, where the inverted cone-shaped PE–PEG stabilizes a mixture of cone-shaped lipids.

## 5 Conclusions

Based on the results we obtained from our simulations, we found out that (A) CHSa stabilizes the DOPE lipid membrane by increasing the hydrophilicity of the bilayer surface, (B) when CHSa changes to CHS by pH reduction, DOPE bilayers are destabilized due to a reduction in bilayer hydrophilicity and a reduction in the area per lipid, and (C) PEG stabilizes DOPE bilayers into the lamellar phase by increasing the area per lipid through penetration into the bilayer, which is the main focus of our study.
